# Medical students’ reactions to an experience-based learning model of clinical education

**DOI:** 10.1007/s40037-013-0061-4

**Published:** 2013-05-03

**Authors:** Alexandra Hay, Sarah Smithson, Karen Mann, Tim Dornan

**Affiliations:** 1 Northwest Deanery, Three Piccadilly Place, Manchester, M1 3BN UK; 2 University of Manchester Medical School, Manchester, M13 9PT UK; 3 Clinical Research Centre, Dalhousie University, 5849 University Avenue, Halifax, NS B3H 4H7 Canada; 4 Department of Educational Development and Research, Maastricht University, P.O. Box 616, 6200 MD Maastricht, the Netherlands

**Keywords:** Clerkship education, Experience based learning, Qualitative analysis

## Abstract

An experience-based learning (ExBL) model proposes: Medical students learn in workplaces by ‘supported participation’; affects are an important dimension of support; many learning outcomes are affective; supported participation influences students’ professional identity development. The purpose of the study was to check how the model, which is the product of a series of earlier research studies, aligned with students’ experiences, akin to the ‘member checking’ stage of a qualitative research project. In three group discussions, a researcher explained ExBL to 19 junior clinical students, who discussed how it corresponded with their experiences of clinical learning and were given a written précis of it to take away. One to 3 weeks later, they wrote 500-word reflective pieces relating to their subsequent experiences with ExBL. Four researchers conducted a qualitative analysis. Having found many instances of responses ‘resonating’ to the model, the authors systematically identified and coded respondents’ ‘resonances’ to define how they aligned with their experiences. 120 resonances were identified. Seventy (58 %) were positive experiences and 50 (42 %) negative ones. Salient experiences were triggered by the learning environment in 115 instances (96 %) and by learners themselves in 5 instances (4 %), consistent with a strong effect of environment on learning processes. Affective support was apparent in 129 of 203 statements (64 %) of resonances and 118 learning outcomes (58 %) were also affective. ExBL aligns with medical students’ experiences of clinical learning. Subject to further research, these findings suggest ExBL could be used to support the preparation of faculty and students for workplace learning.

## Introduction

Learning in clinical settings is essential for medical students’ development into doctors [1, 2]. The nature of medicine and what is expected of practising professionals are changing, however, and medical education has to evolve if it is to serve students well [3–5]. Traditional conceptualizations of apprenticeship, in particular, conflict with some realities of modern clinical workplaces, which include: Short hospital attachments and many-to-many student–teacher relationships; fast-paced rotations between specialities [6]; extremely busy clinical learning environments; and learning collaboratively in inter-professional groups rather than from a single senior doctor [7, 8]. New understandings from the learning sciences suggest traditional models of education overlook many opportunities to support and enhance learning in clinical settings [7, 9–11]. They highlight, also, that affective elements of learning deserve more attention because scientific ‘ways of knowing’ have, traditionally, predominated over them. New education theory emphasizes pragmatic as well as theoretical knowledge and highlights the wide variety of types of knowledge used by practitioners [12]. Workplaces remain important learning environments [1, 13], because they provide opportunities to learn skills for later practice. To make best use of them, learners must take an active part in their own education, set goals, and monitor their progress toward those goals [14]. Self-regulation skills and an ability to learn effectively from experience are now regarded as critical elements of professional competence [1, 14, 15].

Identity formation is increasingly seen as a key outcome of workplace learning [16]. Communities of practice theory (COP) [10], which is one of the most often quoted theories in relation to clinical workplace learning [17], places great emphasis on identity development. According to COP, learners find meaning in experiences gained by participating in practice. By being exposed to a wide variety of experiences, they develop their professional identities. Identity, according to Wenger [10], is a broad construct, which includes knowledge, skills, a sense of professional self, and a wide range of affects. COP theory is relevant to contemporary clinical contexts because it focuses more on learners as members of social groups than as individuals, which reflects the way we now learn and work. It was not developed in relation to clinical education but we have developed a theoretical model of experience-based learning (ExBL), which links the key constructs of COP to aspects of medical students’ learning. Described in greater detail in the Methods, ExBL was developed by conducting research [18–20], which identified how learning outcomes resulted from processes in learning environments, which were enabled by certain conditions. The idea behind developing the model was to change our understanding of clerkship learning from a ‘black box’, whose contents are obscure, to an explicit statement of conditions, processes, and outcomes, which could help train faculty and students to optimize learning, and could contribute to curriculum design.

The purpose of this research was analogous to the member checking stage of grounded theory research, where an emerging theory is offered back to representatives of a research population of interest. Member checking does not rule a theory in or out, but gives an indication of how it aligns with people’s everyday experiences. The research question, then, was: How does an ExBL model align with students’ experiences of clinical learning? The approach was qualitative.

## Methods

This research was approved by the Committee on the Ethics of Research on Human Beings of the University of Manchester (Ref. 10422).

### Conceptual orientation

The epistemological stance of the research was constructionist, according to which ‘truth’ is relative and theory is valuable in so far as it provides useful insights into the experiential world. The constructionist stance made it appropriate to derive sensitizing concepts from a theory and use them to analyze qualitative data, and to use the transaction between data and theory to refine the theory. The research had a conceptual orientation towards COP, as has been explained in the Introduction, and towards ExBL.

**Fig. 1:**
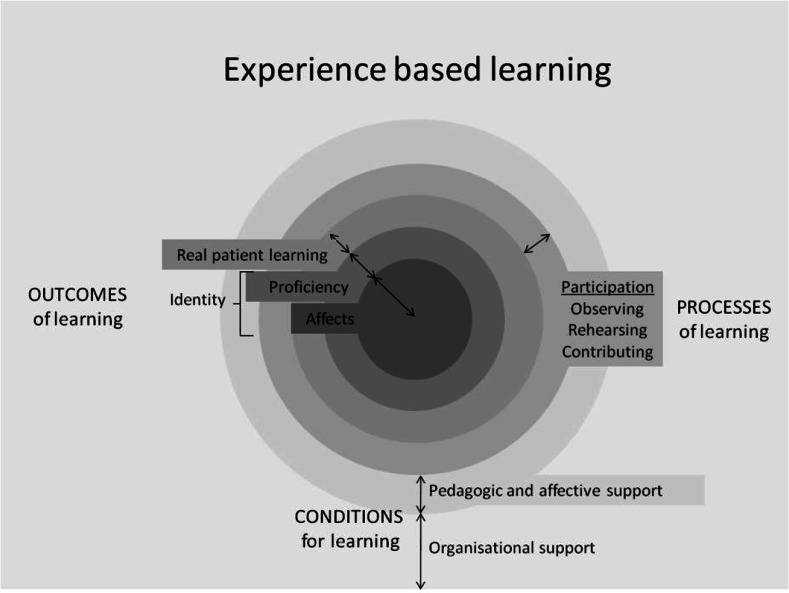
Explanation of the experience-based learning (ExBL) model as given to participating students. Within conditions of organizational support at institutional and placement level, and pedagogic/affective support at the placement and interactional levels, learners participate in practice by observing, rehearsing, or contributing to practice, which results in real patient learning and construction of professional identity, whose two interrelated components are proficiency in workplace activities and affects

The latter (Fig. 1) considers the conditions, mechanisms, and outcomes of ‘authentic’ clinical learning, that is to say learning from real patient experience rather than simulation. ExBL proposes that the key mechanism by which students learn is *participation* in clinical activities, which may take one of three forms: *observing*, *rehearsing*, or *contributing* to patient care. Participation results in *real patient learning* (immediately at the point of contact between student and patient), *proficiencies* (demonstrable practical skills, knowledge and attitudes) and *affects.* Affects include: Motivation, legitimacy, sense of belonging, and sense of professional selves. As in COP, proficiencies and affects together constitute *professional identity*. Participation occurs optimally in conditions of support, which is of three types: *pedagogic support* (supportive teaching), *affective support* (acknowledging emotional aspects of teaching and learning) and *organizational support* (which facilitates both pedagogic and affective support).

### Setting

The Manchester Medical School undergraduate curriculum uses problem-based learning as its main educational method and provides patient contact in all 5 years of the programme, increasing from 4 days/year in years 1–2 to whole-time workplace learning in years 3–5. Its >2,000 students are distributed across four teaching hospital sites with their associated district general hospitals and community healthcare providers. The study was conducted in Central Manchester Foundation Trust (CMFT), which is one of those teaching hospitals.

### Participants

Third-year students, who are at the end of their first year of whole-time workplace learning, were chosen for the study because they have some clerkship experience but still need to develop their workplace learning skills, having only recently transitioned from the pre-clinical to the clinical phase of the programme.

#### Recruitment of student volunteers

An announcement was posted on the medical undergraduate learning management system summarizing the study. It was reinforced by a message delivered by a text messaging system. All students who responded positively were forwarded a document detailing the requirements of participation and they were invited to confirm their participation. With Ethics Committee approval, only verbal consent was obtained. Volunteers were not reimbursed but they were offered:

- Free refreshments on the day of their focus group
- The opportunity to include the 500-word written pieces, whose production was supported by participation in the study, in their learning portfolios
- A certificate of participation, also to be included in their portfolios, upon receipt of the written piece.

### Study design

The study had two stages. In the first, participants attended discussions in groups of 6–7 people lasting 1–2 h at which AH (herself a medical student) presented the ExBL theoretical model and then facilitated a group discussion. She invited participants to think how their prior experiences related to ExBL, elicited their views, asked them to comment, and invited them to ask questions or add ideas of their own. The presentation followed the structure of ExBL and a handout presenting and explaining ExBL (Fig. 1) was distributed but there was no other topic guide so the discussions could be free-ranging. The facilitator’s position as a senior medical student inevitably affected how participants responded but we judged that her understanding of participants’ situations made her a more suitable facilitator than a neutral third party (who would not so well understand medical students’ lifeworlds and be able to explore what they said) or a member of staff (with whom participants would be in a dependent relationship). Discussions were audio-recorded, transcribed verbatim, and anonymized. One to 3 weeks later, participants were asked to submit an anonymized 500-word (minimum) piece of writing in which they reflected on their clinical experiences before and/or after the group discussion, linking them to ExBL.

### Data analysis

All researchers independently read all transcripts and reflective pieces, exchanged interpretations verbally and by email, and met repeatedly to discuss and synthesize the evolving interpretation. Textual material, both from the group discussions and reflective pieces, showed rich and varied ‘resonance’ with the model. It was deemed consistent with the constructionist methodological orientation to identify all such resonances (as well as searching for dissonances) and code several features of each of them: the trigger of the experience described, who initiated it (student or teacher), and the outcome. Many episodes had more than one triggering cause and/or more than one outcome so the number of causes/outcomes in the narrative that follows exceeds the number of resonances. Data were coded using NVivo software, subcoded to the main categories described above, then analyzed using a constant comparative qualitative approach. Quotations in the text are labelled to indicate their source (e.g., FG1-3 indicates focus group 1, participant 3) and the number of instances is shown as numbers in parentheses. We chose to write primarily about the affective outcomes because they were greater in number and are less well represented in the medical education literature [21] than proficiencies (knowledge and skills), which are very well described.

## Results

Nineteen students (13 women and 6 men) participated in the group discussions and 17 (90 %) also provided written submissions. Transcripts of those materials contained 120 resonances (more than one from every respondent), 70 (58 %) of which were positive experiences and 50 (42 %) of which were negative experiences. To illustrate a positive resonance, here is how a respondent described affective support in the form of a welcoming learning environment and enthusiastic teachers, which increased their motivation: ‘*We felt we were a lot more welcomed on vascular surgery and I’ve certainly felt a lot more motivated with them and the junior doctors have been great, really wanting to teach you… and you kind of feel like you want to go in, be taught*’ (FG1-3). A typical negative experience was students allocated to a ward being ‘*ignored for 7* *weeks*’ (FG2-9) and having a sense of being unwelcome and undervalued.

### Outcomes

Because an experience could have more than one outcome, there were 163 outcomes, 112 (69 %) positive outcomes and 51 (31 %) negative outcomes.

#### Practical learning

Forty-five ‘resonances’ coded to this theme of which 34 (76 %) were positive and 11 (24 %) were negative. For reasons explained in the Methods, these are not described here in greater detail, but they were entirely in keeping with the types of knowledge and skills that students would be expected to acquire in clerkships.

#### Affective learning

##### Motivation

Twenty-two of 39 outcomes (56 %) were positive, for example: ‘*occasions when someone has been very willing to teach me, let me take part… I have enjoyed myself and… come away thinking how good my day has been, that I have learnt a lot and am therefore looking forward to getting back and learning more*’ (W13-3). Seventeen outcomes (44 %) were negative, such as: ‘*it can be quite difficult to completely keep yourself motivated… to keep encouraging yourself to continue… if you’re put down constantly with no encouragement it’d be quite easy to feel like ‘what’s the point’ and give up’* (FG2-22).

##### Legitimacy and sense of belonging

There were 16 (80 %) positive and 4 (20 %) negative instances of legitimacy as an outcome. A positive example was: ‘*you actually felt like you belonged and you didn’t feel like you were on the fringes, you actually felt like you were doing something to help’ (FG3*-*7)* A negative one was being made to feel like *‘an outsider’* (FG3-12).

##### Sense of professional self

Fourteen of 18 outcomes (78 %) were positive ones concerning learners’ sense of self as doctors-to-be, for example: ‘*there are definitely some doctors that make you feel like a professional yourself… if I’m treated like a professional with responsibilities I’m more likely to practise to be like that’* (FG1-17). Four (22 %) were negative, such as: ‘*because we’re always told ‘you’re just medical students you can’t pretend that you’re doctors’… I’m more working towards the identity of a proficient medical student rather than a doctor’* (FG2-30).

##### Other affective outcomes

Twelve reported outcomes were increased confidence and three were reduced confidence. Ten concerned being satisfied and four being dissatisfied. The remaining 12 affective outcomes included building relationships and feeling overwhelmed, rewarded, frustrated or encouraged.

### Conditions

One hundred and fifteen experiences (96 %) were triggered by people or situations in respondents’ learning environments and just five were caused by respondents themselves. Because most of those 120 experiences exemplified more than one type of support, there were 203 instances, 129 (64 %) of which were classified as affective support, 59 (29 %) of which were pedagogic support, and 15 (7 %) or which were organizational support.

#### Affective support

Eighty-one statements (63 %) were positive and 48 (37 %) were negative. Thirty-five concerned being welcomed, which had a positive effect such as: ‘*I really enjoyed my first firm… and have credited this… to the welcome that the staff on this ward gave us*’ (W2-1). An unwelcoming environment left students feeling ‘*like I’m giving more trouble just by being an extra person there… you actually feel like you don’t want to go*’ (FG2-6). Thirty statements described teachers’ enthusiasm—in 19 of them, teachers’ enthusiasm led to positive experiences, such as: ‘*they were always really willing for me to come with them. It just made such a difference*’ (FG2-18). Eleven statements described a lack of enthusiasm, which caused students to learn far less: ‘*you don’t get anything from it*’ (FG3-8). Twenty-six statements concerned the friendliness of staff. In 24 positive instances, it was ‘*not necessarily the formality*’ but ‘*the approachability*’ (FG1-12) that causes students to learn. Friendliness inspired confidence—‘*this creates a relaxed and enjoyable atmosphere for learning and I find that I am far more willing to participate and get involved in these situations*’ (W7-3) - and made respondents feel more motivated—*you’re more likely to go and impress them then by reading up on a topic and searching them out*’ (FG1-8). Unfriendliness, described in two statements, made respondents ‘*less likely to volunteer to perform procedures or practice communicating with patients’* (W14-1). Similarly, supervisors behaving supportively (8 statements) facilitated student learning: ‘*you were getting involved in things and you knew you were supported if you needed to ask for help but they were happy to let you go and try things*’ (FG3-2), whereas unsupportive behaviour (2 statements) caused unfavourable outcomes: ‘*I was told not to say anything during the consultations… my GP did not provide the support and encouragement suitable for me to become more involved*’ (W15-1).

Students valued being respected (5 statements) and ‘*appreciated as a team member… not just an added student*’ (FG2-10), but when the opposite was true (12 statements) students’ sense of worth suffered: For example: ‘*some doctors… make you feel like a professional yourself and some… make you feel like a school*-*child… if I’m treated like a student, sort of a child, I behave a bit like that*’ (FG1-16). Being abused and humiliated (9 statements) divided opinion, some respondents asserting that such ‘*old*-*school*’ teaching meant ‘*you learn a lot… fair enough*’ (FG2-19) whereas others felt their learning suffered, being ‘*scared to then go and ask something*’ (FG1-15) and feeling that this style of teaching was ‘*unnecessary*’.

#### Pedagogic support

There were 44 positive and 15 negative statements about pedagogic support. Helping respondents participate in workplace activities was cited in 30 statements. These were both in a positive light (23 statements): ‘*we’ve had one GP who was really good… I’ve actually been asked to go off on my own, clerk in a patient and do all that. You feel involved because he writes exactly what you’ve taken from the history in the notes, so you contribute at every level and I found that really good*’ (FG3-2), and a negative light (7 statements): ‘*it was just me and my clinical partner and we were ignored for 7* *weeks*’ (FG2-9). The importance of instruction (10 statements) and observation and feedback (8 statements) was stated, with respondents claiming that ‘*some of the best learning experiences I have had [are] when I have firstly observed, then I have been able to perform the skill myself, and afterwards had feedback on this*’ (W3-4). A lack of supervision (1 statement) resulted in negative outcomes such as decreased motivation as ‘*there’s no leader… no*-*one to really bounce ideas off… there wasn’t real learning process… it’s actually been really frustrating*’ (FG2-27). Students described modelling their behaviour on that of their supervisors in 11 statements. Positive modelling (4 statements)—‘*something you’ve seen that you think is good and want to take up when you finally become a doctor*’ (FG1-19)—was outweighed by instances of negative modelling (7 statements) in which ‘*when watching doctors interact with patients I’ve thought ‘I don’t want to do that’… I don’t want to treat a patient like that’* (FG1-18).

#### Organizational support

The 15 statements exemplifying organizational supported included: Planning and preparing teaching—‘*at different GP practices, especially the ones where they are very organized… I think they’ve maybe thought more about what they need to teach you*’ (FG1-22); and communication issues regarding cancelling or re-organizing teaching sessions, which often frustrated students and impacted upon their senses of professional self. Here, for example, a student expresses the negative affect they experienced when a disorganized teacher cancelled teaching at short notice: ‘*We’re expected to be professional but then at the same time people aren’t treating us professionally back… obviously you understand that doctors are busy but it’d still be nice to be treated with some respect*’ (FG3-17). Another organizational issue was arranging cover for missing tutors and choosing appropriate placements and/or tutors ‘*that have the time to teach you and… have enough patients that can be seen… rather than just putting students with anyone, then hearing the feedback and saying “well, they weren’t that great but we’ll get another group there”’* (FG3-20).

When a respondent described initiating an experience, it was by: Asking questions, asking for help, or asking for teaching—‘*you then ask the F1* *s to go over everything, they’d teach you how you could present it better…you learn how doctors do it in actual notes*’ (FG2-4); taking the initiative and being proactive with regards to learning opportunities: ‘*I’d be happy to go into wards and do things because I know I’ll be able to do something that would help and not just taking a history and then forgetting about it afterwards*’(FG2-8); and organizing or communicating directly with medical staff. One respondent said direct communication ‘*would contribute to their… identity and… may encourage confidence in the student to request teaching, ask more questions and benefit more from their time*’ (W10-1).

## Discussion

### Principal findings and meaning

The answer to the question ‘*How does an ExBL model align with students’ experiences of clinical learning?*’ is that respondents were able to give many examples of how their workplace learning experiences fitted the model. The fidelity of the model to respondents’ experiences was further supported by statements like ‘*the conditions of learning really affect the processes of it because if you don’t have a consultant who will let you be part of the clinic and let you take histories then you don’t have a chance to move from the observing role to the contributing role*’ (FG2e-1). Whilst the study design made it likely respondents would identify elements of ExBL in their experiences, replicate them in their written accounts, and write positively about the model, some comments were analytical enough to suggest respondents had truly reflected on ExBL. For example, the comments ‘*although you’re made to feel welcome it doesn’t necessarily mean the teaching you’ll have will be that great*’ (FG2e-2) and ‘*if you have someone who is really nice but doesn’t know anything then that’s a bit pointless as well*’ (FG1e-1) show how respondents recognized the distinction between affective and pedagogic support in their own experiences. There was other evidence that students did more than replicate ExBL in an affirmative way to earn points for portfolio learning. Nearly a third of comments were negative ones, describing how the model (which was presented in positive terms) could work in reverse. Also, respondents picked up strongly on the theme of affects, both as conditions for and as outcomes of workplace learning, as captured in the phrase: ‘*for me, positive affective support offered by my teachers does seem to be a key to success*’ (W2e-1). The finding that learning episodes almost always had their origins in the learning environment has to be interpreted cautiously because that is what ExBL predicted. It is striking, however, that ‘*self*-*directedness*’, even when it was described, tended to take the form of eliciting support rather than functioning independently of support.

### Strengths and limitations

The preceding paragraph has highlighted the main threat to the validity of our findings. There is potential, inherent in the study design, for the conditions of the research to predetermine its outcome. That would be a fatal flaw if this were theory-testing research, but that was not its purpose. Rather, the purpose of the research was to find if a working theory resonated well enough with the situations it applied to for further research to be justified. We do not claim the findings to be generalizable, but we do suggest they give sufficient support to justify further testing of the utility of the theory. There are other limitations: The recruitment of self-selected, enthusiastic volunteers; failure of two respondents to complete the study; the relatively short interval between attending the presentation and writing the reflective piece. The methodology has strengths as well as limitations. It has similarities with constructivist grounded theory [22], which is gaining popularity as a way of using prior theory to interpret new empirical findings. By doing so, it is possible to refine theory over time, rather than generate a new grounded theory with each new piece of qualitative research. One final caution is that the numbers of statements in the various categories (for example, the proportion of positive and negative responses) are not intended to be representative of an absolute truth but are offered to give readers a sense of the relative strength of reaction to different elements of ExBL.

### Relation to other publications

In addition to the theories quoted earlier, our findings are consistent with the work of Illeris [23]. He stressed the importance of ‘interaction processes’ between learners and their social environments, and the importance of affective as well as cognitive dimensions of learning. Wilkerson and Doyle [24] recently wrote that ‘*one of the significant changes in… the new millennium is a commitment to help learners improve their skills and potential in regards to the learning experience*’. Whilst Faculty Development is a well-established theme in medical education research [25], learner development (the theme within which our research is located) is an emerging one. Wilkerson and Doyle [24] went on to ask if learner development should be restricted to learners in difficulty or available to all learners. By analogy with elite athletes, they argued that succeeding as well as failing students should be supported in developing learning skills, although efforts to date have been focused primarily on the latter. ExBL is applicable to both succeeding and failing students. Moreover, it differs from interventions that are focused primarily on success in summative assessments. Rather, it addresses the learning skills needed to be effective workplace learners and practitioners. A search of recent accessions to the search engine Google Scholar using the keywords ‘Medical student learner development’ and ‘Medical student study skills’ showed a lack of current research into how best to develop medical students as workplace learners. Teaching (rather than learning) strategies were dominant. Those initiatives that did set out to make students more independent learners were focused on project or non-experiential components of undergraduate medical curricula. An important exception was a review by Murdoch-Eaton and Whittle [26], who wrote of the importance of developing generic learning skills as tools for medical students’ successful lifelong learning. They highlighted the importance of contextualizing learner development to clinical workplace learning, but the means of doing so was beyond the scope of their review.

We have systematically reviewed 168 research publications in the years 2000–2006 inclusive concerning medical students’ ExBL [27]. There were many reports of curriculum innovations, which increased medical students’ immersion in learning environments, clinical teaching, and acquisition of clinical skills. Particularly relevant to this paper were a number of interventions that used education technology [28, 29], learning logs or encounter cards [30, 31], *proformas* to structure patient assessments [32, 33], and the establishment of clinical teaching wards [34] to foster favourable interactions between learners and learning environments. A limitation of many interventions was that they were contextually bound or addressed only very limited learning skills. What the present research adds to them is a generic and comprehensive model of workplace learning that could help make such interventions broader in their scope and provide a framework for evaluating them.

### Implications for research

This research indicates that ExBL has face validity in the eyes of students and provides a rich set of instances of various components of the model. What it does not do is move beyond opening the black box of clerkship learning to show how to train faculty and students, or show how ExBL can contribute to curriculum design. It can, however, help researchers develop testable hypotheses. It might be hypothesized, for example, that strengthening pedagogic, organizational, and affective support would strengthen students’ learning. It might be hypothesized, also, that making students and teachers aware of the affective outcomes of clerkship learning might help them, as members of communities of educational practice, work together to develop students’ identities. ExBL is a complex model, according to which learning outcomes are caused by multiple interacting factors. Randomized controlled trials are of limited value in education research because they test simple rather than complex interventions so we favour using design-based (or action) research methodology to explore the application of ExBL. The main goal of such research, we suggest, would be to find how best to support teachers’ teaching and students’ learning.

## Conclusions

Pending the results of further research, the implications for educational practice are that ExBL provides a model of how medical students’ clerkship learning can be optimized. Traditionally, the emphasis has been on ‘clinical teaching’, which many clinical teachers take to mean transmitting knowledge and skills to students. The focus of ExBL on learning emphasizes whole learning environments of which clinicians are part, rather than just doctors as teachers. It locates agency within learners more than teachers, but gives clinicians important roles in supporting students’ learning. ExBL emphasizes the importance of formally welcoming students to learning environments and introducing them to staff. It emphasizes educational support, particularly when it addresses the affective as well as the cognitive dimension of learning. It argues for students to be treated respectfully as young professionals, rather than ignored or humiliated as was too often the case. It highlights the importance of clinical teachers acting as positive role models, who inspire students towards achieving excellence rather than just doing well enough to pass examinations. The research also argues for the importance of good organization of placements with clear timetables and scheduled opportunities for students to participate in clinical practice, appropriate to their stage of development. To achieve and maintain high standards of clinical placements, a quality development strategy is also required. In parallel with the research reported here, we have validated a measure of the quality of clinical learning environments [21], which makes it possible to measure how well they measure up to the criteria we have proposed and obtain formative, textual feedback for continuing quality development. Finally, students’ positive reactions suggest that using the model could help them see how they learned from experiences, which they had not previously regarded as important.

## Essentials

- Medical students learn by supported participation in the activities of clinical workplaces.
- Positive emotions towards students and their learning are an important dimension of support.
- Many student learning outcomes are also emotional.
- Learning theory can be used to help students and teachers understand how they learn in clinical workplaces.

## References

[1] Cooke M, Irby DM, O’Brien BC. Educating physicians: a call for reform of medical school and residency. San Francisco: Jossey Bass; 2010.

[2] Morris C, Blaney D, Swanwick T (2010). Work-based learning, Chapter 5. Understanding medical education: evidence, theory and practice.

[3] General Medical Council (2009). Tomorrow’s doctors.

[4] Frank JR, Danoff D (2007). The CanMEDS initiative: implementing an outcomes-based framework of physician competencies. Med Teach.

[5] Frank JR, Snell LS, ten Cate O (2010). Competency-based medical education: theory to practice. Med Teach.

[6] Holmboe E, Ginsburg S, Bernabeo E (2011). The rotational approach to medical education: time to confront our assumptions?. Med Educ.

[7] Bleakley A (2006). Broadening conceptions of learning in medical education: the message from team working. Med Educ.

[8] Swanwick T (2005). Informal learning in postgraduate medical education: from cognitivism to ‘culturism’. Med Educ.

[9] Lave J, Wenger E (1991). Situated learning: legitimate peripheral participation.

[10] Wenger E (1998). Communities of practice: learning, meaning, and identity.

[11] Mann K (2011). Theoretical perspectives and pedagogy: past experience and future possibilities. Med Educ.

[12] Eraut M, Mallock M, Cairns L, Evans K, O’Connor BN (2011). How researching learning at work can lead to tools for enhancing learning. The Sage handbook of workplace learning.

[13] Billett S (2002). Toward a workplace pedagogy: guidance, participation, and engagement. Adult Educ Q.

[14] Spencer J, Cantillon P, Wood D (2010). Learning and teaching in the clinical environment. ABC of learning and teaching in medicine.

[15] White CB, Gruppen LD, Swanwick T (2010). Self-regulated learning in medical education, Chapter 9. Understanding medical education: evidence, theory and practice.

[16] Monrouxe LV (2010). Identity, identification and medical education: why should we care?. Med Educ.

[17] Dornan T, Mann K, Scherpbier A, Spencer J, Dornan T, Mann K, Scherpbier A, Spencer J (2010). Introduction. Medical education: theory and practice.

[18] Dornan T, Boshuizen H, King N (2007). Experience-based learning: a model linking the processes and outcomes of medical students’ workplace learning. Med Educ.

[19] Dornan T, Scherpbier A, Boshuizen H (2009). Supporting medical students’ workplace learning: experience-based learning (ExBL). Clin Teach.

[20] Dornan T, Muijtjens A, Graham J, Scherpbier A. Manchester clinical placement index (MCPI). Conditions for medical students’ learning in hospital and community placements. Adv Health Sci Educ Theory Pract. 2011; (Epub ahead of print). 10.1007/s10459-011-9344-x PMC3490061 22234383

[21] McNaughton N (2013). Discourses of emotion within medical education: the ever-present absence. Med Educ.

[22] Charmaz K (2004). Premises, principles, and practices in qualitative research: revisiting the foundations. Qual Health Res.

[23] Illeris K (2003). Workplace learning and learning theory. J Workplace Learn..

[24] Wilkerson L, Doyle L, Dornan T, Mann K, Scherpbier A, Spencer J (2011). Developing teachers and developing learners, Chapter 19. Medical education: theory and practice.

[25] Steinert Y, Mann K, Centeno A (2006). A systematic review of faculty development initiatives designed to improve teaching effectiveness in medical education: BEME Guide No. 8. Med Teach.

[26] Murdoch-Eaton D, Whittle S (2012). Generic skills in medical education: developing the tools for successful lifelong learning. Med Educ.

[27] Dornan T, Tan N, Boshuizen H, et al. Experience-based learning (ExBL). Realist synthesis of the conditions, processes, and outcomes of medical students’ workplace learning (In revision).

[28] Dornan T, Hadfield J, Brown M, Bohuizen H, Scherpbier A (2005). How can medical students’ learn in a self-directed way in the clinical environment? Design-based research. Med Educ..

[29] Leung GM, Johnston JM, Tin KYK (2003). Randomised controlled trial of clinical decision support tools to improve learning of evidence based medicine in medical students. BMJ.

[30] Greenberg LW (2004). Medical students’ perceptions of feedback in a busy ambulatory setting: a descriptive study using a clinical encounter card. South Med J.

[31] Coates WC, Gendy MS, Gill AM (2003). Emergency medicine subinternship: can we provide a standard clinical experience?. Acad Emerg Med.

[32] Penn MA, Smucker W, Logue E (2001). Functional and attitudinal outcomes of teaching functional assessment to medical students. Educ Gerontol.

[33] Shah MN, Heppard B, Medina-Walpole A, Clark NS, McCann R (2005). Emergency medicine management of the geriatric patient: an educational program for medical students. J Am Geriatr Soc..

[34] Ponzer S, Hylin U, Kusoffsky A (2004). Interprofessional training in the context of clinical practice: goals and students’ perceptions on clinical education wards. Med Educ.

